# Supplementary material for: Evaluations of CRC2631 toxicity, tumor colonization, and genetic stability in the TRAMP prostate cancer model
Source: Oncotarget. 2020 Nov 3;11(44):3943–58. doi: 10.18632/oncotarget.27769 (PMC7646835; doi:10.18632/oncotarget.27769)
Supplement: Supplementary file 1 [file oncotarget-11-3943-s001.pdf]

Evaluations of CRC2631 toxicity, tumor colonization, and genetic stability in the TRAMP prostate cancer model

SUPPLEMENTARY MATERIALS

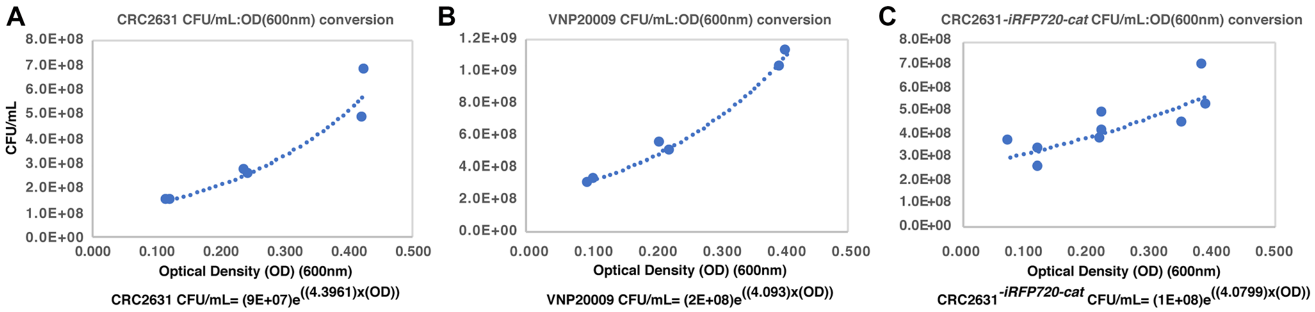

Supplementary Figure 1: Determination of optical density conversion equations. Best fit curve equations (OD to viable cells/mL) of (**A**) CRC2631, (**B**) VNP20009, and (**C**) CRC2631$^{-iRFP720-cat}$ independent clonal populations suspended in PBS at three different 600 nm optical densities (OD) after growth for 24 h in liquid culture and viable cells/mL determined by plating dilution series of each culture on plates containing appropriate selective antibiotics (See Materials and Methods) and enumerated after 30 h incubation at 37°C.

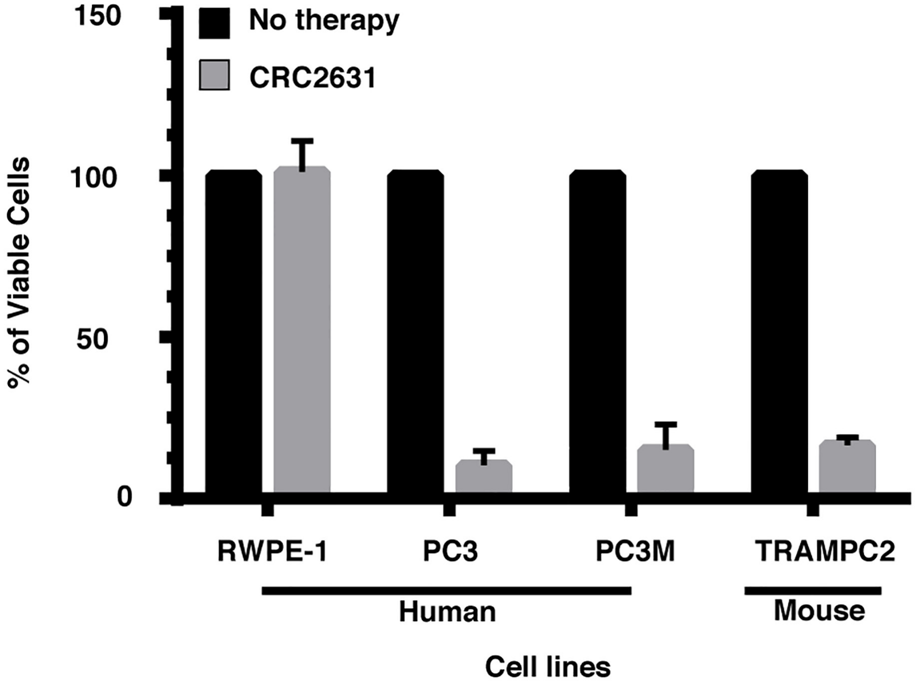

Supplementary Figure 2: CRC2631 specifically targets human and mouse prostate cancer cells. Human benign prostate (RWPE-1), prostate cancer and murine cancer cells (10^4) were treated with 10^4 CFU of CRC2631 for 4 h at 37°C and then washed. Cell viability was assessed using an MTT assay. Results represent the mean ± SD of three trials performed in triplicate.

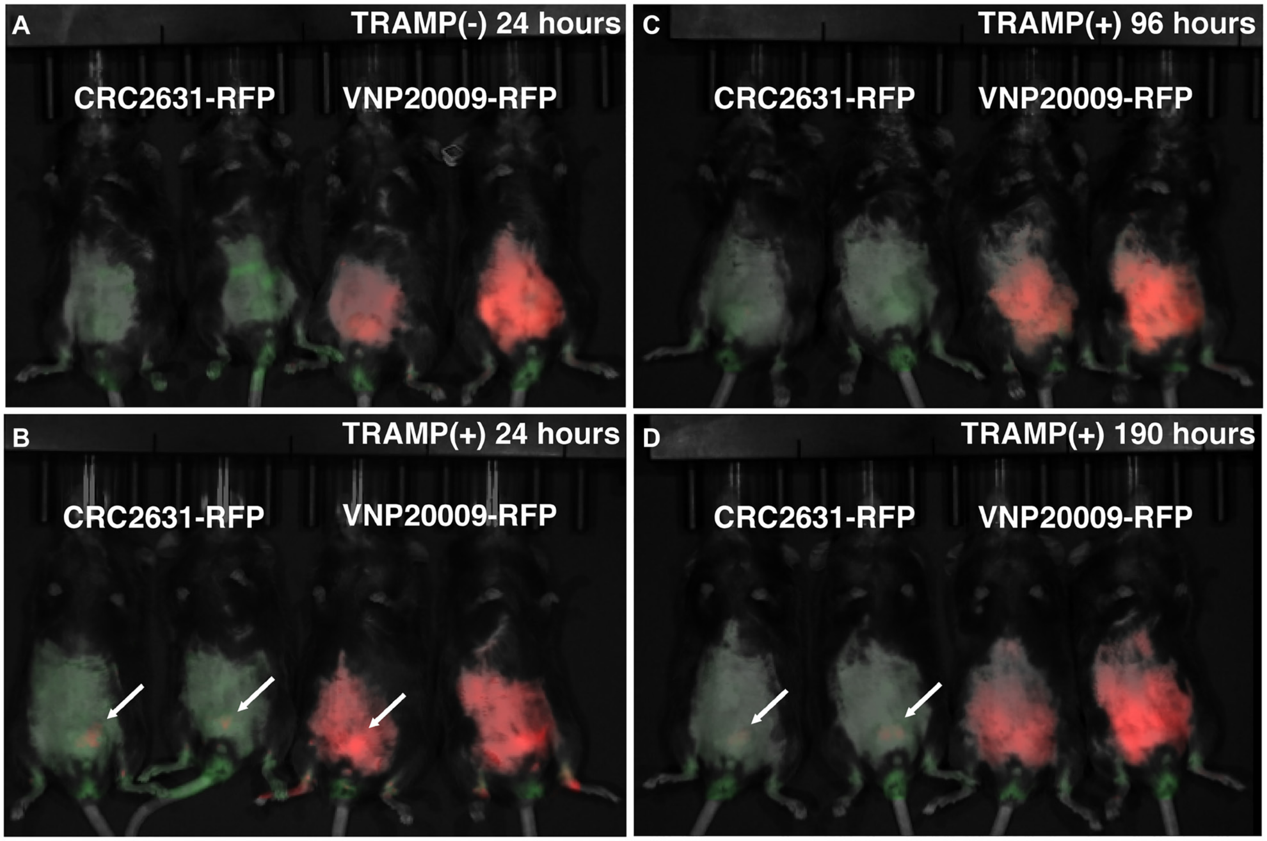

Supplementary Figure 3: Comparative CRC2631 and VNP20009 biodistribution in B6 TRAMP animals. Qualitative localization and persistence of CRC2631 and VNP20009 expressing mCherry red fluorescent protein (RFP) in (**A**) B6 TRAMP (–) or (**B–D**) B6 TRAMP (+) mice bearing primary prostate tumors. Mice (*N=2*) were IP treated with 1×10^6 CFU of CRC2631 or VNP20009 expressing mCherry red fluorescent protein (RFP). Using an IVIS *in vivo* fluorescent imaging system, living mice were scanned at (B) 24, (C) 96, and (D) 190 hours post injection to detect CRC2631 or VNP20009 associated mCherry RFP signal. Red = CRC2631 or VNP20009 associated mCherry signal. Green = tissue autofluorescence. (A) CRC2631 does not persist after 24 hours in B6 TRAMP (–) mice. (B) CRC2631 successfully colonizes the primary prostate tumor (arrows) at 24hpi in B6 TRAMP (+) mice. (C) CRC2631 mCherry signal becomes undetectable at 96hpi, but (D) re-emerges at 190hpi, demonstrating persistence in the B6 TRAMP (+) primary prostate tumor model.

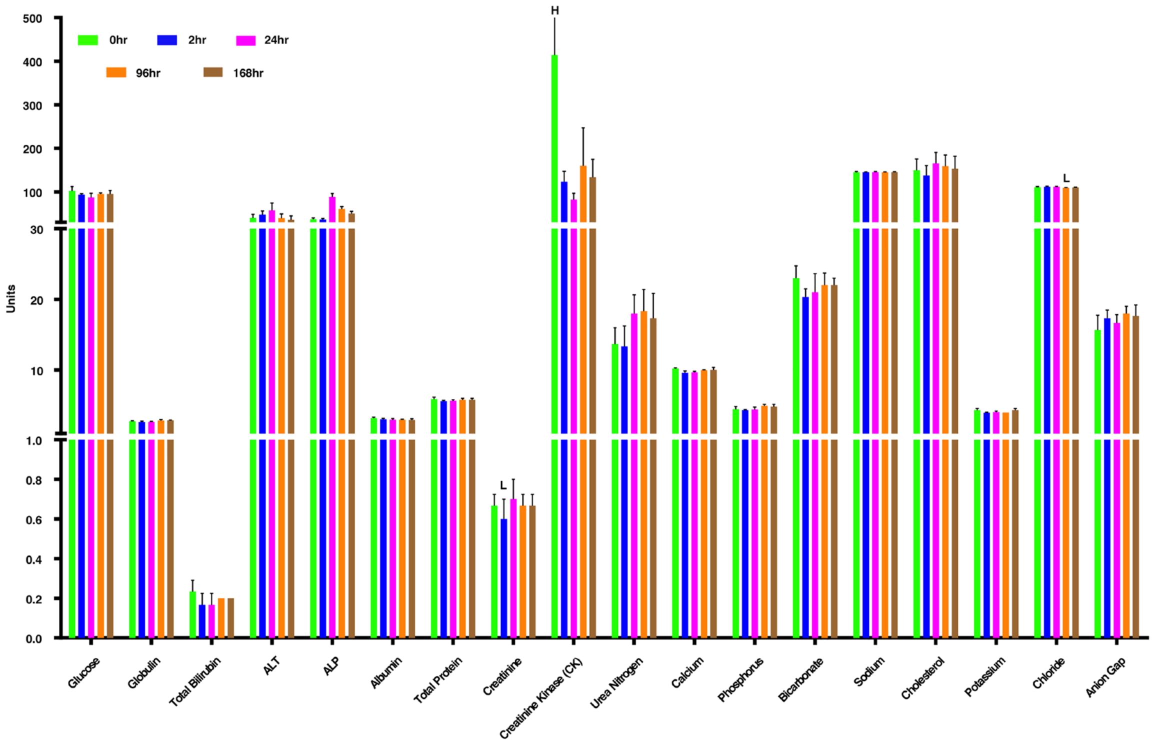

Supplementary Figure 4: Toxicological assessment of CRC2631 in canine models. Three 13-month-old male beagles were IV administered 4×10^6 CRC2631 and plasma samples collected at 0, 2, 24, 96, and 168 h time points. A small animal Maxi Panel was performed to evaluate pathological response to CRC2631 injection. The chart shows the mean levels of plasma chemistry components in the three dogs to identify significant pathologies in organ tissue or metabolic function. All panels that included mean results outside of calibrated normal ranges (L = Low, H = High) are shown. Mean levels of creatinine below normal range at two hours post CRC2631 injection was not significantly different from initial levels ($p < 0.374$). One dog exhibited high levels of creatinine kinase (CK) before injection of CRC2631 but CK levels were within normal range from 2–168 hours post injection and mean CK level changes from pre-injection to post-injection was not significantly different at 2 h ($p < 0.372$), 24 h ($p < 0.316$), 96 h ($p < 0.436$), or 168 h ($p < 0.389$). Mean chloride levels at 96 hpi were below normal range but this was not significantly different from initial chloride levels ($p < 0.230$). Chemistry panels indicate no significant pathologies in organ tissue or metabolic function as a result of IV CRC2631 injections into dogs.

Supplementary Table 1: Bacterial strains, mouse models, and cell lines

Bacteria	Genotype	Reference
LT2	Wild-type *Salmonella enterica* serovar Typhimurium strain.	(McClelland 2001) PMID: 11677609
CRC1674	Derived from LT2 strain, *hisD2550* rpoS. Archived in room temperature agar stab 1958, recovered Nov 1998.	(Sutton 2000) PMID: 10913067
CRC2631	CRC 1674 *aroA551*::Tn10(Tet^R) Δ*rfaH* Δ*thyA*::pKD4 (Kan^R)	(Choe 2014) PMID: 24987088
CRC2636	CRC 2631 pRSET-mCherry	(Choe 2014) PMID: 24987088
VNP20009	*Salmonella enterica* serovar Typhimurium 14028 (YS72 hyperinvasive, xyl$^-$) Δ*purI* Δ*msbB*	(Pawelek 1997) PMID: 9377566, (Clairmont 2000) PMID:10837181
CRC263$^{1iRFP720\text{-}cat}$	CRC 1674 *aroA551*::Tn10(Tet^R) Δ*rfaH* Δ*thyA*::P_{tac}-*iRFP720cat*(Cam^R)	This study
**Mouse**	**Genotype**	**Reference**
B6 TRAMP	C57BL/6-Tg(TRAMP)8247Ng/J (Jax Laboratories)	(Gingrich 1996) PMID: 8797572
B6FVB TRAMP	C57BL/6-Tg(TRAMP)8247Ng/J (Jax Laboratories) x FvBNHsd (Envigo)	(Gingrich 1997) PMID: 9354422
**Cell Lines**	**Genotype**	**Reference**
PC3	Human prostate cancer cells; derived from metastatic site: bone	(Kaighn 1979) PMID: 447482
PC3M	PC3 variant with increased metastatic frequency	(Stephenson 1992) PMID: 1378502
RWPE1	Human epithelial prostate cells	(Bello 1997) PMID: 9214605
TRAMP-C2	Mouse epithelial prostate adenocarcinoma cells	(Foster 1997) PMID: 9269988
